# Inflammation biomarkers are associated with the incidence of cardiovascular disease: a meta-analysis

**DOI:** 10.3389/fcvm.2023.1175174

**Published:** 2023-07-07

**Authors:** Yifei Liu, Suzhen Guan, Haiming Xu, Na Zhang, Min Huang, Zhihong Liu

**Affiliations:** ^1^ School of Public Health, Ningxia Medical University, Yinchuan, China; ^2^ Key Laboratory of Environmental Factors and Chronic Disease Control, Ningxia Medical University, Yinchuan, Ningxia, China

**Keywords:** fibrinogen, interleukin-6, C-reactive protein, galectin-3, cardiovascular disease, meta-analysis

## Abstract

**Background:**

Inflammation is a risk factor for cardiovascular disease (CVD), and particular inflammatory parameters can be used to predict the incidence of CVD. The aim of this study was to assess the association between fibrinogen (FIB), interleukin-6 (IL-6), C-reactive protein (CRP) and galectin-3 (Gal-3) and the risk of cardiovascular disease using meta-analysis.

**Methods:**

PubMed, Embase, Scopus, and Web of Science databases were searched with the appropriate strategies to identify observational studies relevant to this meta-analysis. A random-effects model was used to combine inflammation factor-associated outcomes and cardiovascular disease outcomes, except in the case of galectin-3, where a fixed-effects model was used because of less heterogeneity. Location, age, type of cardiovascular disease, and sample size factors were used to explore heterogeneity in stratification and metaregression for subgroup analysis. A case-by-case literature exclusion approach was used for sensitivity analysis. The funnel plot and Begg's test were combined to assess publication bias.

**Results:**

Thirty-three papers out of 11,456 were screened for inclusion in the analysis. Four inflammation biomarkers were significantly associated with the development of CVD: FIB (OR: 1.21, 95% CI: 1.15–1.27, *P* < 0.001; HR: 1.04, 95% CI: 1.00–1.07, *P *< 0.05), IL-6 (HR: 1.16, 95% CI: 1.10–1.22, *P* < 0.001), CRP (OR: 1.25, 95% CI: 1.15–1.35, *P* < 0.001; HR: 1.20, 95% CI: 1.14–1.25, *P* < 0.001) and Gal-3 (HR: 1.09, 95% CI: 1.05–1.14, *P* < 0.001). Location factors help explain the source of heterogeneity, and there is publication bias in the Gal-3 related literature.

**Conclusion:**

Taken together, the current research evidence suggests that high levels of fibrinogen, interleukin-6, C-reactive protein and galectin-3 are risk factors for cardiovascular disease and can be used as biomarkers to predict the development of cardiovascular disease to some extent.

**Systematic Review Registration:**

https://www.crd.york.ac.uk/PROSPERO, identifier: CRD42023391844.

## Introduction

Cardiovascular disease (CVD) is the leading cause of death and disability worldwide (1). Prevalent cases of total CVD in 204 countries and territories worldwide nearly doubled from 271 million [95% uncertainty interval (UI): 257–285 million] in 1990 to 523 million (95% UI: 497–550 million) in 2019 and are an important contributor to the heavy disease burden in the world (2). In terms of developmental mechanisms, inflammation is not only the basis for the pathogenesis of atherosclerosis but also a common cause of cardiovascular disease (3); thus, certain inflammatory factors may act as biomarkers to predict the risk of cardiovascular disease.

Epidemiological studies have revealed an independent positive link between elevated plasma fibrinogen (FIB) levels and CVD, as well as a positive relationship with several established CVD risk factors (4, 5). In addition, many other factors are associated with subclinical atherosclerotic heart disease (6). Galectin-3 (Gal-3), a potential biomarker of cardiovascular inflammation, promotes the secretion of other proinflammatory factors, such as interleukin-6 (IL-6), in a dose-dependent manner by activating macrophages and is significantly and rapidly expressed in a variety of diseases, including cancer, diabetes, and heart disease (7, 8). IL-6 plays a key role as an upstream cytokine in the propagation of the inflammatory response downstream of atherosclerosis, and the high production of IL-6 stimulates hepatocytes to generate C-reactive protein (CRP), which further amplifies the inflammatory response (9). Inflammation and atherosclerosis are essential features of the pre-CVD condition. Although the results of most studies suggest that inflammatory factors appear to predict cardiovascular events, there are still investigations that yield nonsignificant or even negative correlations for specific subtypes of CVD (10–18). In addition, prevention of CVD is a key component, yet current meta-analyses have focused on clinical prognosis and mortality in CVD patients (19–22), lacking an integrated discussion of the predictive value of inflammatory factors in the development of CVD, and even fewer articles have included consideration of multiple biomarkers simultaneously for direct comparison. Therefore, this study aimed to assess the relationship between these four inflammation biomarkers (FIB, IL-6, CRP, and Gal-3) and the risk of first occurrence of CVD events by performing a meta-analysis of previously published observational studies and, thereby, providing better estimates of the odds ratios.

## Methods

### Search strategies

This meta-analysis is registered on PROSPERO (https://www.crd.york.ac.uk/PROSPERO) with the registration ID CRD42023391844. In databases (EMBASE) (all fields), PubMed (all fields), Web of Science (topic), and Scopus (article title, abstract and keywords), the search strategy “((FIB) OR (fibrinogen) OR (IL-6) OR (interleukin-6) OR (CRP) OR (c reactive protein) OR (Gal-3) OR (galectin-3)) AND (incidence rate) AND (cardiovascular disease)” was used to search for all publications included up to 30 May 2023. The search does not use any filters or restrictions. References of related articles were also manually indexed to ensure maximum comprehensiveness for literature inclusion. All citations were imported into NoteExpress 3.7.0 to remove duplicates.

### Study selection criteria

This meta-analysis involved articles investigating the association between four inflammation markers (FIB, IL-6, CRP, and Gal-3) and the incidence of common CVD (including heart failure, myocardial infarction, coronary artery disease, stroke, coronary heart disease, atrial fibrillation.) selected according to the following specific inclusion and exclusion criteria.

The inclusion criteria were (1) observational studies (including prospective cohort, retrospective cohort, and case‒control); (2) Inflammation factors investigated included one or more of FIB, IL-6, CRP, Gal-3, and an endpoint of first cardiovascular disease; and (3) study data contained hazard ratio (HR)/relative risk (RR)/odds ratio (OR) with 95% confidence intervals (CI) per standard deviation (SD) increase in log-transformed biomarker level.

The exclusion criteria were (1) non-English works of literature; (2) generally nonhealthy adult population (based on history of certain diseases and current diseases or population treated with interventions such as surgery); (3) sample size <500; and (4) when multiple studies assessed identical factors and endpoints across the same group of participants, only the latest was included.

### Data extraction and quality evaluation

The extraction of crucial data and the quality assessment of the study were performed independently by two investigators to ensure that data collection was as accurate and objective as possible. Any disagreements were resolved through further deliberation and intervention efforts involving a third investigator. The results of the maximum adjustment model were used for the selection of the ending indicator. The details and characteristics derived and recorded among all eligible full texts are listed below: (1) study location; (2) participant characteristics (age, sex, sample size); (3) study period; (4) study cohort; (5) type of cardiovascular disease; (6) OR/RR/HR with 95% CI; (7) covariates in the maximum-adjusted model. The Newcastle Ottawa Scale (NOS), developed for case‒control and cohort studies, was used to evaluate quality; the scale ranges from 0 to 9, with 7 and above indicating very good quality, 4–6 indicating fairly good quality, and 4 and below indicating poor quality (23, 24).

### Statistical analysis

The correlation between biomarkers and the risk of CVD was commonly reported as OR/RR/HR values for multiple comparison forms. Studies in which the values were reported by comparing linear biomarker doubling to incident CVD or the 4th tertile of biomarkers compared to the bottom tertile were not considered, and only articles with a 1-SD log-biomarker increase were included. Thus, although total surveys of the same type were not enrolled, comparability of outcome indicators was ensured while introduction of errors by data estimation transformation was avoided. In this meta-analysis, we analyzed studies reporting HR separately, and studies reporting RR and OR were directly combined and transformed into OR for analysis (25, 26). Statistical heterogeneity was assessed with the *I*^2^ statistic and the Cochran *Q* test, where a *P*-value of <0.05 was considered significant for heterogeneity. Pooled results were calculated using random-effects models if *I*^2 ^> 50%, which indicated a substantial degree of heterogeneity across studies, or using a fixed-effects model otherwise (27). We performed subgroup analysis and meta-regression to assess the impact of variables such as region, age, CVD type, and sample size on the pooled effects and heterogeneity. Sensitivity analysis was used to explore whether the presence of certain studies altered the pooled results significantly by removing each study one at a time. Potential publication bias was evaluated collectively with the visual inspection funnel plot and Begg's test (28, 29). Stata (Version 17; MP-64) software was used for the meta-analysis and statistical analysis.

## Results

### Literature search

**Figure 1:**
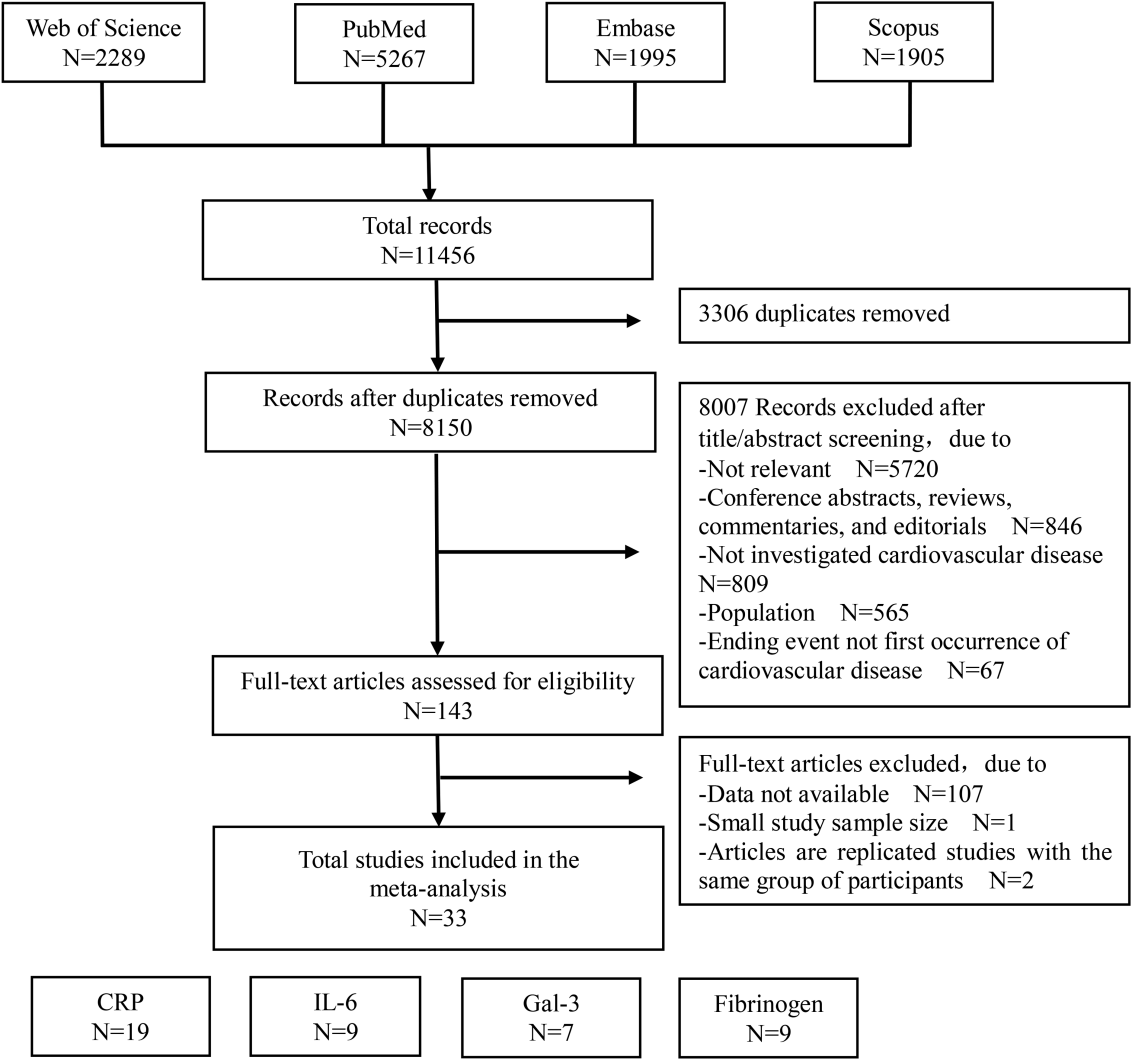
Flow-chart of literature search for meta-analysis.

The literature search and eligible study selection process is shown in Figure 1. A total of 11,456 papers from the Web of Science, PubMed, Embase, and Scopus databases were retrieved by the targeted search strategy, and 33 were finally included in this meta-analysis after removal of duplicates, browsing of abstracts, and refinement of the full text. We estimated that for each inflammation biomarker, there were 7–19 articles that included an evaluation of the biomarker's capacity to predict the risk of cardiovascular disease.

### Study characteristics and quality evaluation

Table 1 summarizes the major information and quality assessment of the included observational studies. The research sites covered European countries such as England, France and Germany (11, 13, 16, 18, 30–38), as well as the United States (10, 12, 15, 39–52), Canada (17) and Japan (53). The research sample size ranged from 564 to 40,656 and was generally adjusted for multiple covariates common to CVD, such as sex, age, blood pressure, smoking, diabetes, BMI, and cholesterol. NOS values of 6–9 in the observational study indicate good quality.

**Table 1:** Study characteristics and quality evaluation.

Reference	Study location	Sample size	Age (years)	Male (%)	Study period	Study cohort	Biomarker	Outcome	Maximum adjusted model RR/HR/OR (95% CI)	Covariates in the maximum-adjusted model	Quality of study (Score 0–9)
Smith 1997	United Kingdom	1,592	64.4 ± 0.2	47.4	1988–1993	Edinburgh Artery Study	FIB	MI	RR: 1.04 (0.89–1.22)	Age, sex, systolic blood pressure, LDL-cholesterol, cigarette smoking, and baseline disease	7
								Stroke	RR: 1.52 (1.17–1.98)		
								CVD	RR: 1.15 (1.02–1.29)		
Scarabin 1998	France and Northern Ireland	10,500	55.0 ± 2.0	100.0	1991–1993	Prospective Epidemiological Study of Myocardial Infarction (PRIME Study)	FIB	CVD	OR: 1.26 (1.17–1.36)	Field center, age, smoking, BMI, waist-to-hip,hypertension, HDL cholesterol LDL-cbolesterol triglycerides and diabetes	7
Koenig 1999	Germany	936	45–64	100.0	1984–1992	Monitoring Trends and Determinants in Cardiovascular Disease Augsburg Cohort Study (MONICA)	CRP	CHD	HR: 1.50 (1.14–1.97)	Age, smoking	8
Scarabin 2003	France and Northern Ireland	10,500	55.0 ± 2.0	100.0	1991–1993	PRIME Study (French participants)	FIB	CHD	RR: 1.26 (1.03–1.60)	Age, BMI, systolic blood pressure, TC, HDL, diabetes, smoking status	6
						PRIME Study (Northern Ireland participants)			RR: 1.42 (1.06–2.16)		
Cesari 2003	USA	2,225	74.0 ± 2.8	44.6	1997–2004	Health, Aging, and Body Composition (Health ABC study)	CRP	CHD	RR: 1.11 (0.96–1.29)	Age, gender, race, smoking, diabetes, hypertension, BMI, HDL cholesterol, triglyceride, and albumin	9
								Stroke	RR: 1.18 (0.91–1.53)		
								HF	RR: 1.48 (1.23–1.78)		
							IL-6	CHD	RR: 1.27 (1.10–1.48)		
								Stroke	RR: 1.45 (1.12–1.86)		
								HF	RR: 1.72 (1.40–2.12)		
Vasan 2003	USA	732	78.4 ± 4.5	33.3	1992–1994	Framingham Heart Study (FHS)	IL-6	HF	HR: 1.36 (1.06–1.74)	Age, sex, diabetes, systolic blood pressure, hypertension treatment, smoking status, BMI, total cholesterol/HDL, valve disease, prevalent atrial fibrillation, prevalent cardiovascular disease, and ECG-LVH	9
Engström 2006	Sweden	6,075	46.8 ± 3.7	100.0	1974–1984	A screening programme is approved and funded by Malmö's health services authority	FIB	Stroke	RR: 1.13 (0.99–1.29)	Age, BMI, diabetes, systolic blood pressure, physical activity, antihypertensive medication, current smoking, tobacco consumption, cholesterol, high alcohol consumption, log triglycerides, angina,occupation, marital status	7
Jeppesen 2008	Denmark	2,357	41–71	49.5	1982–1991	Monitoring trends and determinants in cardiovascular disease project (MONICA)	CRP	CVD	HR: 1.33 (1.14–1.55)	Age, sex, smoking habit and total cholestero,waist circumference,levels of triglycerides, high-density lipoprotein-cholesterol, systolic and diastolic blood pressures, and level of physical activity	7
Davidson 2009	Canada	1,794	46.3 ± 18.3	49.9	1995–1999	The Canadian Nova Scotia Health Survey (NSHS95)	CRP	CHD	HR: 1.28 (1.04–1.57)	Sex and framingham risk score	6
							IL-6		1.07 (0.90–1.27)		
Schnabel 2009	USA	2,863	61 ± 9	45	1998–2001	The Framingham Offspring cohort	FIB	AF	HR: 0.94 (0.79–1.12)	Age, sex, smoking, systolic blood pressure, hypertension treatment, body mass index, diabetes, alcohol consumption, electrocardiographic left ventricular hypertrophy, auscultatory valvular heart disease, myocardial infarction, and heart failure	6
							CRP		HR: 1.05 (0.87–1.26)		
							IL-6		1.08 (0.91–1.29)		
Smith 2010	Sweden	5,187	57.6 ± 5.9	41.0	1991–1994	MDCS (Malmö Diet and Cancer Study)Cardiovascular Cohort (MDCCC)	CRP	HF	HR: 1.57 (1.28–1.94)	Age, sex, systolic blood pressure, diastolic blood pressure, use of antihypertensive treatment, body mass index, low-density lipoprotein, high-density lipoprotein, current smoking,history of diabetes mellitus, and history of myocardial infarction	8
								AF	HR: 1.18 (1.03–1.34)		
Chei 2011	Japan	13,314	40–85	51.0	1985–2000	Circulatory Risk in Communities Study (CIRCS)	CRP	Stroke	OR: 1.17 (1.01–1.35)	Systolic blood pressure, antihypertensive medication use, body mass index, alcohol intake category, cigarette smoking status, serum total cholesterol levels, log-transformed tryglyceride levels, and serum glucose category as well as matching for sex, age, community, year of serum stored, and fasting status	7
Schnabel 2013	USA	3,035	61.0 ± 9.0	47.0	N/A	Framingham Heart Study (FHS)	CRP	CVD	HR: 1.21 (1.06–1.39)	Age, sex, smoking, systolic blood pressure, hypertension treatment, total/HDL cholesterol, body mass index, and diabetes	7
							FIB		HR: 1.18 (1.03–1.36)		
							IL-6		HR: 1.16 (1.01–1.32)		
Daniels 2014	USA	1,397	70.0 ± 11.0	35.8	1992–2009	Rancho Bernardo Study	Gal-3	CHD	HR: 1.09 (0.92–1.30)	Age, sex, diabetes, hypertension, current smoking, systolic blood pressure, total cholesterol, HDL, estimated GFR, BMI, log_10_NT-proBNP	6
Yin 2014	USA	942	65.0 ± 9.0	69.0	1991–2008	Framingham Heart Study (FHS)	CRP	MI	OR: 1.87 (1.09–3.19)	age, sex, current smoking status, statin use, systolic blood pressure, hypertension treatment status, total cholesterol, high-density lipoprotein–cholesterol, diabetes mellitus status, and body mass index	7
								CVD	OR: 1.38 (1.13–1.69)		
Ho 2014	USA	3,306	58.0 ± 9.0	46.0	1995–1998	The Framingham Offspring cohort	Gal-3	AF	HR: 1.13 (0.95–1.36)	Age, sex, clinical risk factors, alcohol, eGFR, BNP, CRP, echocardiographic parameters	7
Jagodzinski 2015	Finland	8,444	25–74	50.8	1997–2012	FINRISK97 Study	Gal-3	MI	HR: 1.06 (0.94–1.20)	Region of Finland, HDL and total cholesterol, systolic blood pressure, antihypertensive medication, smoking, prevalent diabetes, prevalent valvular heart disease, eGFR, galectin-3, NT-proBNP	6
								Stroke	HR: 1.06 (0.94–1.18)		
								HF	HR: 1.09 (0.99–1.19)		
Seven 2015	Denmark	6,502	45.9 ± 7.9	48.1	N/A	Inter99 Study	CRP	Stroke	HR: 1.08 (0.88–1.33)	Sex, age, intervention group,total cholesterol, HDL-cholesterol, smoking status, systolic blood pressure, treatment for hypertension, baseline diabetes,BMI, HOMA-IR, eGFR, adiponectin, leptin	6
								IHD	HR: 1.15 (1.00–1.32)		
Appiah 2015	USA	10,601	59.6 ± 5.6	43.0	1993–2012	Atherosclerosis Risk in Communities study (ARIC)	FIB	CHD	HR: 1.00 (0.94–1.06)	Age, sex, race, ARIC center,education, smoking, alcohol intake, sports index, systolic blood pressure, body mass index, use of antihypertensive medications, diabetes mellitus, cholesterol medication, high-density cholesterol, and total cholesterol,total fibrinogen,high sensitivity C-reactive protein	7
								Stroke	HR: 0.97 (0.87–1.07)		
								HF	HR: 1.05 (0.99–1.12)		
AbouEzzeddine 2016	USA	1,614	54–71	47.0	1997–2009	Epidemiologic research in Olmsted County	Gal-3	HF	HR: 1.20 (1.03–1.41)	Age, sex, BMI, eGFR, hypertension, systolic blood pressure, CAD, diabetes mellitus, total cholesterol, HDL and smoking history	9
Dawood 2016	USA	25,841	64.5 ± 9.5	45.3	2003–2007	REasons for Geographic And Racial Differences in Stroke Study (REGARDS)	CRP	Stroke	HR: 1.06 (1.01–1.12)	Age, sex, race and socioeconomic status, hypertension, diabetes mellitus, congestive heart failure and warfarin/aspirin use	6
Appiah 2016	USA	5,888	≥65	90.0	1992–2013	CHS population-based cohort	FIB	CHD	HR: 1.02 (0.95–1.10)	Age, sex, race, years of education and CHS center, smoking status, alcohol intake, physical activity, systolic blood pressure, BMI, antihypertensive medications use, diabetes, cholesterol medication use, HDL cholesterol and total cholestero, total fibrinogen	7
								Stroke	HR: 0.88 (0.77–1.00)		
								HF	HR: 1.00 (0.92–1.08)		
Silverman 2016	USA	6,781	45–84	50.0	2000–2007	Multi-Ethnic Study of Atherosclerosis (MESA)	CRP	HF	HR: 1.17 (0.93–1.46)	Gender, race/ethnicity, socioeconomic status, MESA site	6
							IL-6		HR: 1.32 (0.91–1.93)		
Tunstall 2017	United Kingdom	15,737	49.0 ± 8.3	52.0	1995–2009	Scottish Heart Health Extended Cohort (SHHEC)	CRP	CHD	HR: 1.13 (1.06–1.19)	Use the Best and Extended ASSIGN Models	7
Ghorbani 2018	USA	2,477	57.0 ± 9.0	45.0	1995–2018	Framingham Heart Study (FHS)	Gal-3	CVD	HR: 1.20 (1.02–1.41)	Baseline galectin-3 levels, age, sex, systolic blood pressure, antihypertensive treatment, diabetes, body mass index, smoking, left ventricular hypertrophy, HDL to cholesterol ratio, estimated glomerular filtration rate, prevalent cardiovascular disease	9
								HF	HR: 1.26 (1.00–1.59)		
de Boer 2018	USA	22,756	60.0 ± 13.0	56.9	1989–2002	Cardiovascular Health Study (CHS); Framingham Heart Study (FHS); Multi-Ethnic Study of Atherosclerosis (MESA); Prevention of Renal and Vascular End-stage Disease (PREVEND)	FIB	HF	HR: 1.12 (1.03–1.22)	Age, sex, race/ethnicity, systolic blood pressure, hypertension treatment, body mass index, diabetes, smoking, presence of left ventricular hypertrophy or left bundle branch block, and previous myocardial infarction	9
							IL-6		HR: 1.10 (0.99–1.22)		
							CRP		HR: 1.04 (0.95–1.14)		
							Gal-3		HR: 1.02 (0.93–1.12)		
Subirana 2018	Spain	5,404	35–74	49.4	1995–2005	REGICOR population-cohorts	CRP	CAD	HR: 1.00 (0.76, 1.33)	Systolic blood pressure, diastolic blood pressure, high-density lipoprotein-cholesterol, total cholesterol, diabetes, smoking	6
							IL-6		HR: 1.34 (1.03, 1.74)		
Ho 2018	USA	3,523	62.0 ± 8.0	47.0	1998–2018	Framingham Heart Study (FHS)	CRP	HF	HR: 1.38 (1.19–1.60)	Age, sex, systolic blood pressure, hypertension treatment, diabetes mellitus, body mass index, smoking, total and HDL cholesterol, and history of atrial fibrillation, prevalent myocardial infarction	7
Leening 2018	USA	3,285	50–79	0.0	1994–2005	Women's Health Initiative Observational Study (WHI OS)	CRP	CVD	HR: 1.20 (1.11–1.30)	Age, race/ethnicity, treated and untreated systolic blood pressure, total and HDL cholesterol levels, diabetes mellitus, and smoking status	7
								CHD	HR: 1.21 (1.09–1.34)		
								Stroke	HR: 1.23 (1.11–1.35)		
Magnussen 2019	Europe	40,656	21–99	48.3	N/A	DanMONICA, FINRISK, Moli-sani, Northern Sweden	CRP	HF	Men: HR: 1.31 (1.23–1.41)	Body mass index, systolic blood pressure, antihypertensive medication, total cholesterol, diabetes, daily smoking	8
									Women: HR: 1.10 (1.00–1.20)		
Aguilar 2020	USA	6,538	62.5 ± 5.6	58.1	2011–2013	Atherosclerosis Risk in Communities study (ARIC) visit 5	Gal-3	CHD	HR: 1.33 (0.68–2.60)	Age, sex, race, total cholesterol, high-density lipoprotein cholesterol, systolic blood pressure, antihypertensive medication, current smoking, and diabetes mellitus status except for heart failure, estimated glomerular filtration rate,log N-terminal pro-B-type natriuretic peptide and log high-sensitivity cardiac troponin	7
								Stroke	HR: 1.02 (0.43–2.43)		
								HF	HR: 1.93 (1.15–3.24)		
Fernandez 2020	Sweden	4,469	57.2 ± 5.9	39.5	1991–2016	Malmö Diet and Cancer-Cardiovascular Cohort (MDC-CC)	IL-6	HF	HR: 1.22 (1.10–1.37)	Age, sex,GDF-15, IL-6, ST2, uPAR	6
Dykun 2022	N/A	8,563	64.6 ± 9.3	78.0	N/A	Assessment of Clinical Effects of Cholesteryl Ester Transfer Protein Inhibitor with Evacetra pib in Patients with High-Risk for Vascular Outcomes (ACCELERATE)	CRP	Stroke	HR: 1.32 (1.08–1.62)	Age, race, sex, region, smoking status, body mass index, diabetes, baseline high-sensitivity C-reactive protein, baseline low-density lipoprotein cholesterol, baseline high-density lipoprotein cholesterol, baseline systolic blood pressure, baseline statin use, and treatment group	6
								MI	HR: 1.28 (1.12–1.46)		

IHD, ischemic heart disease; MI, myocardial infarction; CVD, cardiovascular disease; CHD, coronary heart disease; HF, heart failure; CAD, coronary artery disease; AF, atrial fibrillation.

### Association of inflammation markers with the risk of CVD

**Figure 2:**
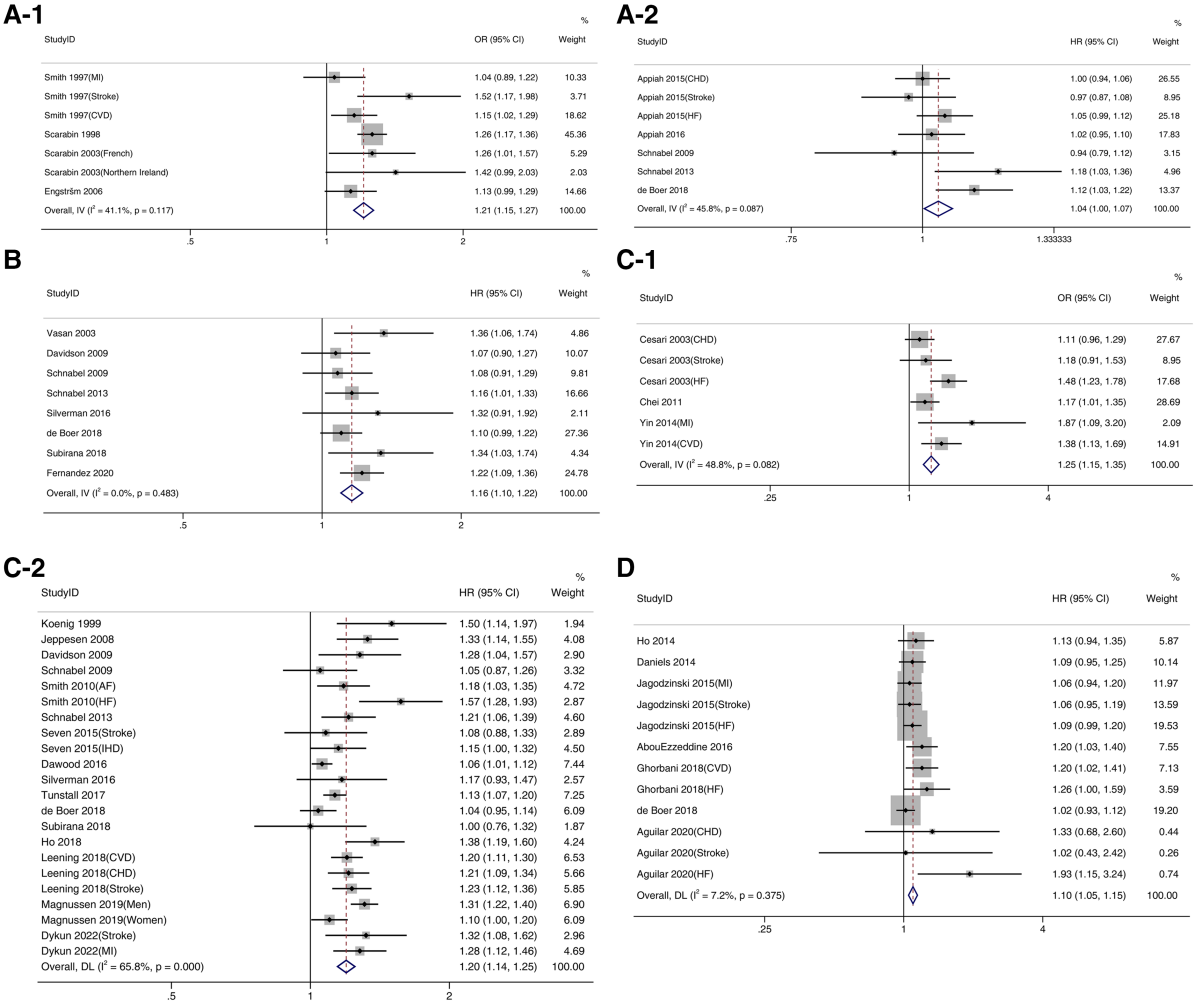
Forest plot of inflammatory factors and incidence of cardiovascular disease.

The results of the meta-analysis are shown in Figure 2 and Table 2, where one-standard deviation increases in the levels of all listed inflammation markers.

**Table 2:** Meta-analysis of inflammation markers and risk of CVD.

Biomarker	Pooled results (95% CI)	Signifificance test (*P*-value)	No. of studies	No. of effffect estimates	*P*-value for Heterogeneity	*I* ^2^
FIB	OR: 1.21 (1.15–1.27)	<0.001	4	7	0.117	41.1%
	HR: 1.04 (1.00–1.07)	0.029	5	7	0.087	45.8%
IL-6	HR: 1.16 (1.10–1.22)	<0.001	8	8	0.483	0.0%
CRP	OR: 1.25 (1.15–1.35)	<0.001	3	6	0.082	48.8%
	HR: 1.20 (1.14–1.25)	<0.001	16	28	<0.001	65.8%
Gal-3	HR: 1.09 (1.05–1.14)	<0.001	7	12	0.375	7.2%

FIB (OR: 1.21, 95% CI: 1.15–1.27, *P* < 0.001, Figure 2A-1; HR: 1.04, 95% CI: 1.00–1.07, *P *< 0.05, Figure 2A-2), IL-6 (HR: 1.16, 95% CI: 1.10–1.22, *P* < 0.001, Figure 2B), CRP (OR: 1.25, 95% CI: 1.15–1.35, *P* < 0.001, Figure 2C-1; HR: 1.20, 95% CI: 1.14–1.25, *P* < 0.001, Figure 2C-2) and Gal-3 (HR: 1.09, 95% CI: 1.0–1.14, *P* < 0.001, Figure 2D) were significantly associated with the risk of developing CVD. There was high heterogeneity in the pooled analysis with CRP reported as HR (*I*^2 ^= 65.8% ≥50.0%, *P* < 0.05).

### Subgroup analysis and metaregression

We performed subgroup analysis based on predefined classification criteria, and the results are shown in Table 3. In most subgroups, high concentrations of FIB, IL-6, CRP, and Gal-3 were associated with an increased risk of CVD, with significantly lower intragroup heterogeneity in more than half of the subgroups. We used metaregression to detect the source of heterogeneity, and the results (Table 4) revealed that the covariate of study location (*P *= 0.004) could help explain heterogeneity in investigations of the biomarker FIB.

**Table 3:** Subgroup analysis of inflammation markers and risk of CVD.

Biomarker	Grouping criteria	Subgroup	Pooled results (95% CI)	Signifificance test (*P*-value)	No. of studies	No. of effffect estimates	*P*-value for Heterogeneity	*I* ^2^
FIB	Location	EUR	1.21 (1.15–1.27)	<0.001	3	4	0.117	41.1%
	Age	<60	1.23 (1.16–1.31)	<0.001	1	3	0.453	0.0%
		≥60	1.15 (1.05–1.26)	0.002	5	7	0.053	66.0%
	CVD type	CVD	1.23 (1.15–1.31)	<0.001	2	2	0.199	39.3%
		MI	1.04 (0.89–1.22)	0.626	1	1	–	–
		Stroke	1.20 (1.07–1.35)	0.003	2	2	0.048	74.3%
		CHD	1.30 (1.08–1.57)	0.006	1	2	0.576	0.0%
	Sample Size	<5,000	1.15 (1.05–1.26)	0.002	1	3	0.053	66.0%
		≥5,000	1.23 (1.16–1.31)	<0.001	3	4	<0.453	0.0%
IL-6	Location	EUR	1.24 (1.12–1.37)	<0.001	2	2	0.518	0.0%
		USA	1.14 (1.06–1.22)	<0.001	5	5	0.491	0.0%
		CAN	1.07 (0.90–1.27)	0.441	1	1	–	–
	Age	<60	1.19 (1.09–1.30)	<0.001	3	3	0.294	18.3%
		≥60	1.14 (1.06–1.22)	<0.001	5	5	0.491	0.0%
	CVD type	CVD	1.16 (1.01–1.32)	0.030	1	1	–	–
		CAD	1.34 (1.03–1.74)	0.029	1	1	–	–
		Stroke	1.45 (1.12–1.86)	0.004	1	1	–	–
		CHD	1.07 (0.90–1.27)	0.441	1	1	–	–
		HF	1.18 (1.10–1.26)	<0.001	4	4	0.297	18.6%
		AF	1.08 (0.91–1.29)	0.387	1	1	–	–
	Sample Size	<5,000	1.17 (1.09–1.25)	<0.001	5	5	0.429	0.0%
		≥5,000	1.14 (1.04–1.25)	0.006	3	3	0.287	19.8%
CRP	Location	EUR	1.21 (1.13–1.30)	<0.001	7	10	0.001	67.5%
		USA	1.16 (1.09–1.24)	<0.001	7	9	0.002	67.4%
		CAN	1.28 (1.04–1.57)	0.019	1	1	–	–
	Age	<60	1.22 (1.13–1.32)	<0.001	7	9	0.026	54.2%
		≥60	1.19 (1.12–1.25)	<0.001	9	13	<0.001	72.6%
	CVD type	CVD	1.22 (1.15–1.30)	<0.001	3	3	0.499	0.0%
		MI	1.28 (1.12–1.46)	<0.001	1	1	–	–
		Stroke	1.15 (1.03–1.29)	0.010	4	4	0.018	70.3%
		CHD	1.20 (1.20–1.31)	<0.001	4	4	0.129	47.1%
		HF	1.23 (1.10–1.38)	<0.001	5	6	<0.001	82.8%
		IHD	1.15 (1.00–1.32)	0.048	1	1	–	–
		CAD	1.00 (0.76–1.33)	>0.999	1	1	–	–
		AF	1.13 (1.02–1.26)	0.022	2	2	0.314	1.4%
	Sample Size	<5,000	1.23 (1.18–1.29)	<0.001	7	9	0.359	9.2%
		≥5,000	1.17 (1.10–1.24)	<0.001	9	13	<0.001	72.5%
Gal-3	Location	EUR	1.07 (1.01–1.14)	0.024	1	3	0.907	0.0%
		USA	1.11 (1.05–1.18)	<0.001	6	9	0.204	27.1%
	Age	<60	1.10 (1.04–1.16)	<0.001	3	6	0.652	0.0%
		≥60	1.08 (1.01–1.16)	0.020	4	6	0.135	40.5%
	CVD type	CVD	1.20 (1.02–1.41)	0.027	1	1	–	–
		MI	1.06 (0.94–1.20)	0.350	1	1	–	–
		Stroke	1.06 (0.95–1.99)	0.316	2	2	0.931	0.0%
		CHD	1.10 (0.96–1.25)	0.161	2	2	0.568	0.0%
		HF	1.09 (1.03–1.16)	0.002	5	5	0.049	58.2%
		AF	1.13 (0.94–1.35)	0.182	1	1	–	–
	Sample Size	<5,000	1.16 (1.08–1.25)	<0.001	6	5	0.782	0.0%
		≥5,000	1.06 (1.01–1.12)	0.017	3	7	0.362	8.8%

**Table 4:** Meta-regression analysis by potential modifier res % residual variation due to heterogeneity.

Biomarker	Covariate	Exp (b)	*P-*value	*I*^2^ res
FIB	Location	1.20 (1.09–1.32)	0.004	41.08%
	Age	0.94 (0.76–1.17)	0.525	41.24%
	CVD type	0.98 (0.91–1.06)	0.555	73.16%
	Sample Size	1.06 (0.85–1.32)	0.525	41.24%
IL-6	Location	1.00 (0.90–1.11)	0.936	7.30%
	Age	0.96 (0.83–1.10)	0.457	0.00%
	CVD type	0.99 (0.95–1.03)	0.516	0.24%
	Sample Size	0.98 (0.84–1.13)	0.705	5.16%
CRP	Location	0.98 (0.93–1.03)	0.338	65.61%
	Age	0.97 (0.88–1.08)	0.591	67.35%
	CVD type	0.99 (0.97–1.01)	0.405	65.11%
	Sample Size	0.94 (0.86–1.00)	0.143	61.82%
Gal-3	Location	1.05 (0.94–1.16)	0.379	10.41%
	Age	0.98 (0.89–1.09)	0.747	14.66%
	CVD type	1.00 (0.96–1.03)	0.766	14.81%
	Sample Size	0.92 (0.89–1.02)	0.090	0.00%

### Sensitivity analysis and publication bias

**Figure 3:**
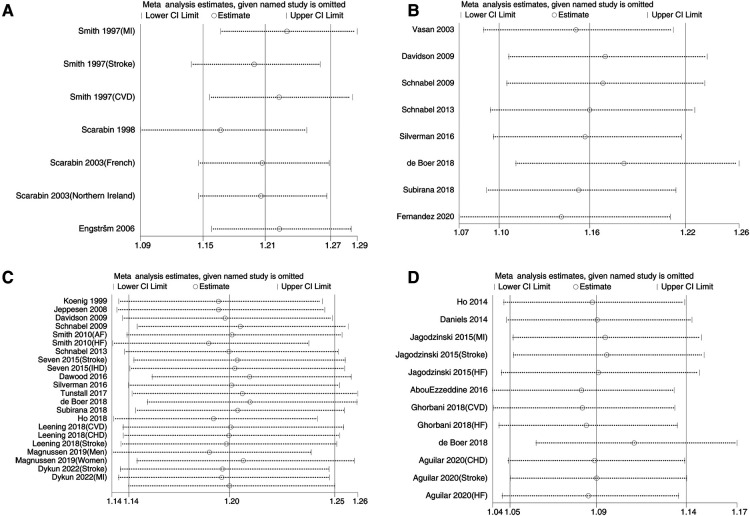
Sensitivity analysis of inflammatory factors and incidence of cardiovascular disease.

**Figure 4:**
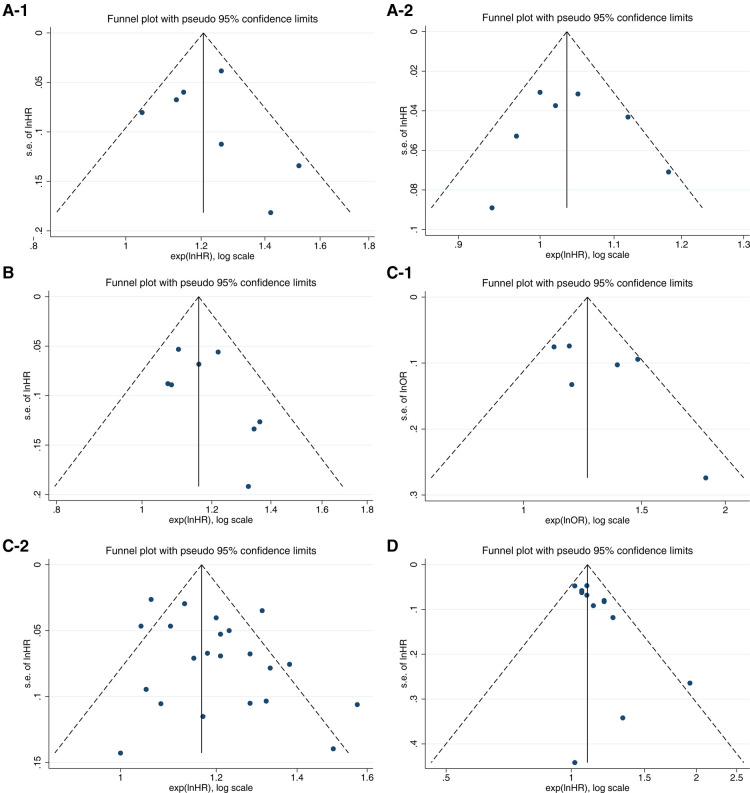
Funnel plots of inflammatory factors and incidence of cardiovascular disease.

Figure 3 shows the sensitivity analysis of the pooled outcomes of the other studies after omitting one study at a time. The combined values of any remaining studies support the conclusion that these inflammation biomarkers are a risk for CVD occurrence, and the small difference between this value and the original effect size suggests that the combined results are not dominated by any one study and are relatively stable and reliable. Figure 4 shows mild asymmetry in the funnel plots of the Gal-3 studies. The results of Begg's test in Table 5 confirm that the Gal-3 related articles were associated with significant publication bias (*P *< 0.05).

**Table 5:** Results of publication bias analysis.

Biomarker	Funnel plot symmetry	Begg's test (*P*-value)
FIB-OR	YES	0.764
FIB-HR	YES	0.764
IL-6	YES	0.266
CRP-OR	YES	0.452
CRP-HR	YES	0.259
Gal-3	NO	0.047

## Discussion

The pooled results of this meta-analysis showed that FIB, IL-6, CRP, and Gal-3 are risk factors for the development of CVD. A juxtaposition of the results showed that the pooled results of the four inflammatory factors were similar and that IL-6 had the best homogeneity of relevant studies. Location helps explain the heterogeneity of the included studies. Sensitivity analysis showed relatively stable combined results.

A number of biomarkers have become a major focus for improving methods of scoring CVD risk. The use of these biomarkers is attractive because they integrate signals from multiple pathophysiologic pathways and have some combined predictive utility (54). The results of the meta-analysis are consistent with previous studies, such as epidemiological investigations showing that for CVD-related diseases, circulating concentrations of FIB are associated with stroke and that their elevated levels predict the incidence of HF (55–57). As the accepted “gold standard,” CRP has been confirmed in numerous studies to be a marker of future CVD risk (58–63). Similar to CRP, levels of IL-6 in populations that appear to be healthy may also be used to predict future vascular risk. This finding was first established in men in 2000 (64), then validated in women (65), and then duplicated in more than 25 prospective epidemiological cohorts throughout the world (66). The findings of this investigation were consistent with those of a meta-analysis by the Emerging Risk Factors Collaborative, which established that a 1 SD rise in log IL-6 was related to a 25% increase in the risk of future vascular events (66). The relationship between the cascade response of IL-6 and CRP may help explain the similar ability of the two to predict the occurrence of CVD in the results of this study (67). Gal-3, as an emerging marker, has attracted increasing interest in recent years. Since 2014, the US Food and Drug Administration has included Gal-3 on the list of validated cardiovascular biomarkers (68). In a large population of 5,805 older adults, low Gal-3 levels were a good negative risk marker regarding the incidence of CVD events observed during short-term follow-up (69). Other factors not included have the potential to be explored as predictive markers, such as the soluble lectin-like oxidized low-density lipoprotein receptor-1 (sLOX-1) that is implicated in atherosclerotic cardiovascular disease (ASCVD) pathogenesis (70). It showed good discrimination in predicting plaque progression in patients with acute coronary syndromes (ACS) (70). sLOX-1 was further confirmed as an early diagnostic marker for ACS (71). Growth differentiation factor-15 (GDF-15) is cytokine involved in the regulation of multiple systems (72). Circulating levels of GDF-15 may be a biomarker of subclinical atherosclerosis in patients with psoriasis (73). In the general population, exploration of relevant markers remains to be further investigated.

A variety of factors can influence the predictive role of inflammation biomarkers of CVD development, including but not limited to those addressed in the exploration of heterogeneity above. Regarding age factors, observational studies on people aged 85 years indicate that a few of the classical risk factors, such as age, sex, systolic blood pressure, total lipoprotein cholesterol, diabetes, and smoking, become blurred or even play an inverse role in predicting cardiovascular disease under the influence of advanced age factors (74, 75). Markers such as C-reactive protein, interleukin 6, fibrinogen, and may help predict CVD status in the elderly population (76, 77). The magnitude of the predictive effect between different inflammation factors on specific CVD subtypes also varies. Any heterogeneity between CVD subtypes implies a search for increasingly specific etiological indicators (78). In a community-based cohort study, elevated plasma Gal-3 levels measured in a middle-aged population were associated not only with the onset of heart failure but also with coronary heart disease, ischemic stroke, and overall mortality (10). A previously published meta-analysis involved examining the relationship between Gal-3 and the incidence of HF, and the subgroup results of the present study were highly consistent in that context (79). Our findings show that IL-6 is one of the strongest inflammation biomarkers for predicting stroke. Indeed IL-6 has been one of the most studied biomarkers of inflammation in stroke patients, especially as a prognostic marker, and as a predictor of stroke risk (80). Similarly, in the case of stroke, CRP levels are rapidly elevated (81), and this acute phase response occurs in the context of widespread inflammation, reflecting the low specificity of CRP elevation. In population-based studies, elevated CRP levels not only increase the risk of stroke (82) but are also associated with poor functional outcome and mortality after stroke (83), and the time points of CRP elevation may reflect different phenomena, with early elevations associated with stroke severity and late elevations associated with post-stroke infection (84). In addition, IL-6 and CRP may be associated with diagnosis, risk stratification and prognosis in patients with acute myocardial infarction (85), and both are also significantly upregulated in acute coronary syndromes (86). There was an association between fibrinogen concentration and CVD, including CAD and stroke incidence, and an increased risk of future myocardial infarction, independent of other CVD and atherosclerotic factors (87, 88). The results of the present study also support this view at the CVD level, and the insignificant results of the subgroup analysis may be due to a bias caused by the small number of studies with the corresponding subgroups. The data in this paper are not grouped by gender, but studies show that women develop CVD later, experience more complications and have worse outcomes than men (89–91). Inflammatory biomarkers such as interleukins and tumor necrosis factor play a role in predisposing CVD risk, and gender-specific differences in CVD risk occurrence are associated with these biomarkers (92). In the Framingham Heart Study, cardiac biomarkers, including CRP, were observed to be substantially higher in premenopausal women than in men, and this difference was attenuated in postmenopausal women who did not receive hormone replacement therapy (93). The more widely supported view regarding the cause of this phenomenon of increased CVD and altered levels of inflammatory factors in women after menopause is that estrogen has an anti-inflammatory effect (94), and therefore, the protective effect of estrogen may be one of the reasons for the differences in biomarker prediction between men and women. A cohort study revealed that the association of some biomarkers with future cardiovascular events was influenced by ethnicity (95); data on CRP, IL-6 and FIB showed that CRP was predictive only in Caucasians, IL-6 was predictive only in African Americans, FIB was predictive in Caucasians, African Americans and Hispanics, and none of the biomarkers predicted CVD in the Chinese population. Despite the fact that Prof. Gu Dongfeng's team has developed a predictive model for assessing CVD in the Chinese population that takes into account differences in the disease spectrum and CVD risk factors compared to other ethnic groups (96), we encourage the continued exploration of the usefulness of novel biomarkers such as Gal-3 in predicting the effect of CVD.

Given modern developments, biomarkers are economical and convenient to measure, so the exploration of biomarkers has strong practical importance. As mentioned earlier, these predictive inflammation biomarkers help identify people at risk for CVD and assess the magnitude of an individual's future CVD risk. Appropriate use can be effective for CVD prevention in the general population. In populations already suffering from CVD, these factors can assist in diagnosis and predict disease prognosis. Nowadays, with the continuous development of anti-inflammatory drugs, certain factors are even indicators for the evaluation of anti-inflammatory effects and targets for treatment. For example, IL-6 has been rapidly gaining attention as a key targeted by the atherosclerosis research community (97). The anti-inflammatory drug ziltivekimab was effective in reducing IL-6 and subsequent hsCRP levels in patients with chronic kidney disease (98). Anti-inflammatory treatment with canakinumab targeting the interleukin-1β (IL-1β) innate immune pathway resulted in lower hsCRP levels and a significantly lower recurrence rate of cardiovascular events in the canakinumab group compared to the placebo group (99). Unlike canakinumab, which selectively inhibits IL-1β, the anti-inflammatory properties of colchicine involve multiple cellular and molecular mechanisms, and the use of small doses of colchicine reduces the composite risk of CV death, spontaneous myocardial infarction, ischemic stroke, or ischemia-driven coronary revascularization in patients with CAD (100).

This study involved pooling evidence from high-quality studies with long durations, large sample sizes, and cardiovascular disease occurrence as an endpoint; furthermore, most of the included studies adjusting for important confounders, including age, sex, BMI, smoking and alcohol consumption, and cholesterol. Multiple inflammatory factors were investigated in this study, and the use of these inflammatory markers in conjunction with each other or in combination with other risk factors may be a way forward in future CVD prediction. For example, in one of the studies, patients in the highest tertile with respect to a specific set of 3 inflammatory markers exhibited an extremely high risk of CVD (39). However, whether the multiple biomarker strategy is superior to the single biomarker strategy still needs further clarification (101). Despite these strengths, it is undeniable that our study has certain limitations. As described in the statistical analysis section, we were unable to include all studies of the same type to ensure reliable data, which inevitably led to some loss and waste of information. Similar to many meta-analyses, the large sample size of this study may have statistically inflated the estimate of heterogeneity (102, 103). In addition, most of the studies conducted in this area were in Europe and the United States, so the lack of findings from Asia, Africa, and Latin America in our included studies suggests a need for further research in the future.

## Conclusion

In conclusion, FIB, IL-6, CRP, and Gal-3 are positively associated with the development of CVD in the general adult population and are risk factors for CVD. Combined consideration of the levels of these inflammatory factor can help in the prediction of future cardiovascular disease.

## Data Availability

The original contributions presented in the study are included in the article, further inquiries can be directed to the corresponding author.
